# Environmentally Relevant Mixture of Pesticides Affect Mobility and DNA Integrity of Early Life Stages of Rainbow Trout (*Oncorhynchus mykiss*)

**DOI:** 10.3390/toxics9080174

**Published:** 2021-07-22

**Authors:** Shannon Weeks Santos, Jérôme Cachot, Bettie Cormier, Nicolas Mazzella, Pierre-Yves Gourves, Christelle Clérandeau, Bénédicte Morin, Patrice Gonzalez

**Affiliations:** 1 CNRS, EPOC, EPHE, University of Bordeaux, UMR 5805, 33600 Bordeaux, France; shannon.wks@gmail.com (S.W.S.); bettie.cormier@u-bordeaux.fr (B.C.); pierre-yves.gourves@u-bordeaux.fr (P.-Y.G.); christelle.clerandeau@u-bordeaux.fr (C.C.); benedicte.morin@u-bordeaux.fr (B.M.); patrice.gonzalez@u-bordeaux.fr (P.G.); 2 National Research Institute of Science and Technology for Environment and Agriculture, 33612 Aix-en-Provence, France; nicolas.mazzella@inrae.fr

**Keywords:** copper, glyphosate, chlorpyrifos, early life stages, rainbow trout, swimming behavior, DNA damage, development

## Abstract

The aim of this study was to analyze the impact of three concentrations of a pesticide mixture on the first development stages of rainbow trout (*Oncorhynchus mykiss*). The mixture was made up of three commonly used pesticides in viticulture: glyphosate (GLY), chlorpyrifos (CPF) and copper sulfate (Cu). Eyed stage embryos were exposed for 3 weeks to three concentrations of the pesticide mixture. Lethal and sub-lethal effects were assessed through a number of phenotypic and molecular endpoints including survival, hatching delay, hatching success, biometry, swimming activity, DNA damage (Comet assay), lipid peroxidation (TBARS), protein carbonyl content and gene expression. Ten target genes involved in antioxidant defenses, DNA repair, mitochondrial metabolism and apoptosis were analyzed using real-time RT-qPCR. No significant increase of mortality, half-hatch, growth defects, TBARS and protein carbonyl contents were observed whatever the pesticide mixture concentration. In contrast, DNA damage and swimming activity were significantly more elevated at the highest pesticide mixture concentration. Gene transcription was up-regulated for genes involved in detoxification (*gst* and *mt1*), DNA repair (*ogg1*), mitochondrial metabolism (*cox1* and 12S), and cholinergic system (*ache*). This study highlighted the induction of adaptive molecular and behavioral responses of rainbow trout larvae when exposed to environmentally realistic concentrations of a mixture of pesticides.

## 1. Introduction

Increased concern about chemical contaminants in natural environments has led to a growth in research aimed at predicting the impacts of these contaminants on ecosystems.

Pesticides are the only kind of chemicals that are purposely re-leased into the environment [1]. Currently, more than 400 chemical compounds are used to treat crops against pests and weeds [2]. Global pesticide usage is calculated at 4.6 million tons per year [1,3]. It has been estimated that 98% of pesticides applied to agricultural crops do not reach their intended target, and could affect terrestrial and aquatic ecosystems. Studies have shown that 40% of worldwide land mass poses a risk in terms of pesticide runoff into rivers and streams [4]. While most ecotoxicological studies focus on individual pesticides, several reports have also confirmed that pesticides are usually found in complex mixtures at low concentrations [2,5], and aquatic organisms are directly exposed to them [6,7,8]. Exposure to pesticide mixtures may trigger additive effects when molecules have similar modes of action and affect the same molecular target, or by independent action when they have dissimilar modes of action affecting the same or different molecular targets, and therefore synergism or antagonism may occur [9].

Because of its anti-microbial and anti-fungal properties, copper (Cu), also listed as a priority substance in the water Framework Directive (WFD) [10], is widely used to protect vineyards from fungal diseases. Given its intensive use for both conventional and organic agriculture, Cu is a widely present pollutant in the environment, and transfer from soils to aquatic ecosystems is likely to occur [11,12]. While it has an essential role in numerous cellular processes, excess Cu generates toxicity by producing reactive oxygen species (ROS) which may cause damages to lipids, proteins and nucleic acids, leading to cell death [13,14]. In a previous study, first stages of rainbow trout were found to be very sensitive to Cu [15]. Indeed, after a 3-week exposure to environmental concentrations of Cu (2 and 20 µg/L), inhibitory effect on hatching and significant induction of malformations were observed. In addition, several genes were down-regulated in Cu-exposed rainbow trout, especially those involved in detoxification (*gst*, *mt1* and *mt2*) and in cell cycle regulation (*p53*).

Glyphosate (GLY) is a broad-spectrum, non-selective and systemic herbicide used in numerous phytosanitary products. It acts as an inhibitor of the 5-enolpyruvyl-shikimate-3-phosphate synthase (EPSPS), an enzyme involved in the shikimic acid pathway that is common in all plants. Because of its extensive use, especially in genetically modified crops [16], high concentrations (ranging from 7.5 to 700 µg/L) have been found in different streams and lakes near agricultural basins [17,18,19]. Several reports have highlighted the sub-lethal effects of GLY on fish in particular on DNA integrity [20,21,22], acetylcholinesterase (AChE) inhibition [23], swimming behavior alterations [24,25], and antioxidant enzyme activities [26,27]. We reported that, following a 3-week embryo-larval exposure to glyphosate (0.1 and 1 mg/L), rainbow trout larvae exhibited reduced head size when compared to control larvae. Additionally, swimming behavior was also affected and exposed larvae had increased mobility compared to non-exposed larvae [28].

Chlorpyrifos (CPF) is an organophosphorus insecticide (OP) which has been widely used in the past by both industrial and private users. However, because of its high toxicity for humans and animals, the use of the product for domestic purposes has been restricted (US EPA), and there is a declining trend in its consumption [3]. Because it is on the list of priority substances for the WFD, its presence in surface and groundwater is closely monitored. While CPF has been detected predominantly in sediments from cultivated areas, low concentrations have also been observed in stream water, where maximum concentrations ranged between 0.06 and 0.45 µg/L in the USA and Argentina, respectively [29,30]. As an OP, CPF affects the nervous system by inhibiting the AChE enzyme [31], disrupting the transmission of nerve impulses through synaptic terminals and impacting essential functions such as respiration and swimming behavior of fish [32,33]. In a recent study, low concentrations of CPF (0.3 and 3 µg/L) did not affect rainbow trout embryonic and larval viabilities. However, sub-lethal effects were observed on mobility of larvae, which was reduced for those exposed to 3 µg/L of CPF compared to control conditions. Low concentrations of CPF also down-regulated genes involved in steroid hormone pathways such as *er-b* and *cyp19a1* [34].

These three compounds (Cu, GLY and CPF), which have differing modes of action and functions, may be applied to crops simultaneously. Given the ease with which they are transported into water bodies through runoff, spray drift, or groundwater, it could be valuable to consider the toxicity of their combined effects, using realistic environmental concentrations, in aquatic organisms. To date, data on the effects of pesticide mixtures remain scarce, particularly relating to the early life stages of fish. To assess the sublethal effects of environmental concentrations of a pesticide mixture, a 3-week exposure was performed on rainbow trout embryos and several developmental and behavioral endpoints were recorded. The endpoints studied included survival, hatching delay, hatching success, morphological anomalies, swimming behavior, genotoxicity (measured by the comet assay), lipid peroxidation and protein carbonyl content, and gene transcription levels. The expression levels of ten genes were selected according to their biological functions in antioxidant defenses (*cat*, *sod*), detoxification (*mt1*, *gst*), mitochondrial metabolism (*cox1*, 12S), cholinergic system (*ache*), DNA repair (*ogg1*, *rad*) or apoptosis (*bax*).

## 2. Materials and Methods

### 2.1. Test Chemicals

Chlorpyrifos-ethyl (CAS No. 2921-88-2) was purchased from ChemService (Merseyside, UK). Copper sulfate (CuSO_4_·H_2_O, CAS No. 7758-99-8, 99.99%) was purchased from Sigma Aldrich (Lyon, France). Glyphosate was purchase as a commercial formulation (available on the market) Roundup^®^GT Max. The active substance of Roundup^®^GT Max is 480 g/L of glyphosate acid, which is equivalent to 588 g/L of potassium salt of glyphosate.

### 2.2. Exposure System

Eyed-stage rainbow trout embryos (*Oncorhynchus mykiss*) were purchased from INRAe-PEIMA (Sizun, FR). Embryos were exposed for 3 weeks from stage 240 DD (Degree Days) to larvae stage 500 DD at 12 ± 0.5 °C. Stock solutions of copper (0.67, 2 and 20 mg/L) and glyphosate (33.3, 100 and 1000 mg/L) were prepared using osmosis water. Stock solutions of chlorpyrifos-ethyl (33.3, 100 and 1000 mg/L) were prepared using DMSO (dimethyl sulfoxide) as a solvent. Each condition (control-solvent mixture conditions) contained 0.0003% DMSO.

Prior embryonic exposure, experimental apparatus units and tanks (1 L in polyethylene terephthalate) were saturated with chlorpyrifos diluted in distilled water for 2 weeks with the studied concentrations for each condition, to ensure saturation of aquaria and avoid dramatic decrease of this compound during exposure. Test solutions were pre-pared by dilution of the stock solutions in a 5 L tank of spring water (dechlorinated) from Laqueuille (4.7 mg/L Ca, 1.8 mg/L Mg, 5.9 mg/L Na, 2.8 mg/L K, 40.3 mg/L HCO_3_^−^, 0.2 mg/L SO_4_^2−^, 0.5 mg/L NO_3_^−^, 7.5 pH, <1.2 mg/L Cl^−^), oxygenated and renewed every two days. The experimental conditions were designed as follows: condition A (solvent control), condition B (0.1 µg/L of CPF + 0.67 µg/L of Cu + 33.3 µg/L of GLY), condition C (0.3 µg/L of CPF + 2 µg/L of Cu + 100 µg/L of GLY) and condition D (3 µg/L of CPF, 20 µg/L of Cu + 1000 µg/L of GLY). Conditions C (3-fold condition B) and D (10-fold condition C), which agree with the medium and highest concentrations in this study, correspond to the lowest and highest concentrations previously studied [15,28]. Each studied condition consisted of three replicates with 75 embryos in one L aquaria. A peristaltic pump (ISMATEC, ISM942) allowed the maintenance of a continuous flow rate (9 mL/min) of contaminated water from tanks into the incubation aquaria. Dissolved oxygen was measured each day with a fiber-optic mini-sensor Fibox 3 (PreSens Precision Sensor, Regensburg, Germany) and data was recorded with OxyView v6.02 software (PreSens Precision Sensor).

### 2.3. Chemical Analysis of Pollutants in Water

Water samples were collected at T_0_ (at the moment of exposure), T_24_ and T_48_ (before water was renewed). Water samples were analyzed to determine Cu concentrations. Water samples of 40 mL for each condition were acidified with 5% of nitric acid (Nitric acid 65%, Fluka). Copper concentrations in water of condition “20 µg/L” and fish samples were analyzed by inductively Coupled Plasma Optic Emission Spectrometry (ICP-OES 720, Agilent Technologies), whereas copper concentrations in water of controls and “2 µg/L” conditions were analyzed using an atomic absorption spectrophotometer (Varian SpectrAA 240Z, Agilent Technologies, Santa Clara, CA, USA). Detection limit (DL) for ICP-OES was 2.26 µg/L Cu^2+^ and for atomic absorption was 0.5 µg/L Cu^2+^. Glyphosate concentration and its main metabolite amino-methyl-phosphonic acid (AMPA) in water samples were analyzed using the method described by [35]. These samples were analyzed using an HPLC-ESI MS (Dionex Ultimate 3000, Thermo Fisher scientific—API 2000 ABSciex). Concentrations were determined with a calibration curve from 1 to 10 µg L^−1^. To ensure accuracy of each analysis, isotope-labeled surrogates was quantified, derivatization blanks were analyzed and quality controls were performed every 10 samples during analysis. Finally, data processing was performed with Analyst V1.6.2. CPF concentrations in water samples were measured by chromato-graphic methods using the Trace GC Ultra Gas Chromatograph (Thermo Fisher Scientific) equipped with an AS-3000 Autosampler (Thermo Fisher Scientific) and coupled to a TSQ Quantum GC Triple Quadrupole (Thermo Fisher Scientific). The limit of quantification was 100 ng/L. The control of the device and the data processing were carried out by the XCalibur software.

### 2.4. Embryo-Toxicity Assay

Embryonic and larval viability was recorded daily, and dead embryos were removed. Embryonic and larval viability denote the number of living individuals compared to the total number of embryos at the start of the experiment or total number of hatching larvae. Hatching rate is calculated by dividing the number of hatched embryos by the total number of embryos at the beginning of the experiment. Duration of development expressed in degree-days (DD) is the duration of embryonic development from fertilization to hatching. At the end of the experiment, 15 larvae per replicate (*n* = 45) were placed individually in a Petri dish with ice water and a few drops of carbonated water to sedate them. Photos of each larva were taken with stereomicroscope (MZ 7.5 Leica) coupled to a camera CCD (DFP420C Leica) and a cold light (Intralux^®^ 4100, Volpi AG, Schlieren, Switzerland). From the photos, total body length, head length and yolk sac area were measured for each larva. Larvae were also observed for the presence of developmental anomalies including edemas, yolk-sac absorption, spinal malformations, craniofacial anomalies, and presence of hemorrhages [15,36].

### 2.5. Swimming Behavior Analyses

Swimming behavior analysis was carried out on 10 larvae per replicate (*n* = 30) at the end of the 3-week exposure. For 30 min, larvae were acclimated in the dark in 6-well microplates containing 3 mL of exposure solution at 12.0 ± 0.5 °C. After acclimation, the microplates were placed in the recording chamber (Daniovision Image Analysis System with Ethovision software version 12.0 Noldus) coupled to a thermoregulatory system set at 12 ± 0.5 °C (Pilot one^®^, Huber). The DanioVision recording chamber includes a white light that can mimic a day/night cycle. Trout larvae were subjected to a light stress with a cycle duration of 30 min including 10 min dark, 10 min light and 10 min dark. An infrared camera in the recording chamber recorded the motion of each larva in response to light stimulation. The Ethovision software recorded the larval velocity every 30 s. Average velocity, total distance swam and time of mobility were determined for each light and dark phase.

### 2.6. Comet Assay with FPG Enzyme

Comet assay was performed on blood cells sampled by decapitation of 6 larvae per replicate (*n* = 18) using a heparinized pipette. Before decapitation, larvae were sedated using iced water and a few drops of carbonated water. Samples were immediately frozen in liquid nitrogen in microtubes with 200 µL of cryo-conservation solution (250 mM sucrose, 40 mM citrate trisodique, 5% DMSO, pH adjusted to 7.6 with nitric acid 1 M) until analysis. To improve the sensibility of the comet as-say, slices were incubated with form-amido-pyrimidine glycosylase (Fpg) enzyme for 30 min at 37 °C, as described by [37]. The comet assay was assessed following the protocol of [36] and [15]. After 20 min of DNA unwind in alkaline solution, electrophoresis was performed with a voltage of 25 V and 300 mA for 20 min. After nuclei separation, slides were stained with 20 µg/mL of ethidium bromide solution, and comet lecture was carried out using an epifluorescence microscope (Olympus BX51) at ×20 equipped with an Olympus U-RFL-T reflected fluorescence system lamp. 100 nuclei per slide were quantified using the Comet Assay IV software (Instrument Perspective LtD). Results are expressed as a percentage of DNA tail.

### 2.7. Biochemical Analysis

#### 2.7.1. Preparation of Supernatant

Pools of 2 larvae (Three pools per replicate, *n* = 9) (approximately 250 mg for one pool) were homogenized in 250 µL of phosphate buffer (0.1 M; pH 7.5; 4 °C) using an UltraTurrax^®^ tissue homogenizer with a potter at 9000 g and 4 °C. The supernatant S9 fraction was obtained after centrifugation at 9000× *g* for 25 min at 4 °C. Each S9 fraction was split up into three tubes for total protein, TBARS and carbonyl protein analysis.

#### 2.7.2. Total Protein

The protein content was determined according to the method described by [38] on the S9 fraction. Measurements were performed using bovine serum albumin as standard, and absorbance was recorded at 750 nm using a spectrophotometer microplate reader (Synergy HT, BioTek).

#### 2.7.3. Lipid Peroxidation (TBARS)

Lipid peroxidation was performed as reported by [39], adjusted for a microplate reader. A volume of 500 µL of a solution containing 20% of butylated hydroxytoluene (BHT) and 20% of trichloroacetic acid (TCA) was added to 500 µL of S9 fraction. The mixture was centrifuged at 9000× *g* for 10 min. Then, 600 µL of the supernatant was added to 120 µL of HCl and 480 µL of TRISbase (25 mM)-TBA (thio-barbituric acid—100 mM) and heated at 80 °C for 15 min. Afterwards, samples were cooled in iced water and mixed. The absorbance of the mixtures was measured using a UV-spectrophotometer (Synergy HT, BioTek) at 530 nm. Results were expressed as nmol of thio-barbituric acid reactive substance (TBARS) equivalents per mg of protein.

#### 2.7.4. Protein Carbonyl Assay

Protein carbonyl content was performed using the procedure of [40]. S9 fraction (500 µL) was added to 50 µL of a solution of streptomycin sulfate (11%)-phosphate buffer (100 mM pH 7.4), mixed and incubated for 15 min at room temperature. Mixtures were centrifuged at 6000× *g* for 10 min and then split up into two tubes. The first one was used as a control and contained 200 µL of supernatant and 800 µL of HCl (2.5 M), and the other was used as a sample, where 200 µL of supernatant was added to 800 µL of DNPH (2.4-dinitrophenylhydrazine 10 Mm), and then left incubated for 1 h. Proteins were precipitated with 20% TCA (trichloroacetic acid), and the formed pellets were washed 3 times by resuspension with 1 mL of ethanol-ethyl acetate (v:v). Pellets were solubilized with 500 µL of 6 M guanidine hydrochloride and centrifuged at 10,000× *g* for 10 min. The measure of carbonyl content was performed with a UV-spectrophotometer (Biotek Synergy HT) at 370 nm. Results are expressed as nanomoles of DNPH incorporated/mg of protein, using the molar absorption coefficient of 22,000 M^−1^cm^−1^.

### 2.8. Gene Expression

At the end of the exposure, 6 larvae per replicate (*n* = 18) were sampled and kept individually in RNA later buffer (Qiagen). Samples were stored at −80 °C.

#### 2.8.1. RNA Extraction

Total RNAs were extracted in whole larvae using the kit SV Total RNA Isolation system” (Promega) following the indications of the provider. Larvae were mixed and homogenized with the MP fastprep^®^-24 (Biorad, 6 m/s, 40 s) using ceramic beads (MP Biomedicals, Lysing Matrix D bulk).

#### 2.8.2. Retro-Transcription of Total RNA into cDNA

The reverse transcription of total purified RNA was performed using the kit “GoScript Reverse Transcription System” (Promega). A mixture of 1 µL of oligo dT (1 µM), 1 µL of hexanucleotides (1 µM) and 10 µL of total purified RNA (1 µg) were mixed and heated for 5 min at 70 °C followed by 5 min at 4 °C with a thermocycler (Eppendorf Mastercycler) to allow primer annealing. Afterwards, 1 µL of dNTP solution (10 mM), 4 µL of activity buffer, 1.5 µL of MgCl2 (25 mM), 1 µL of reverse transcriptase (1 U/µL) and 0.5 µL of RNAsine were added. Reverse transcription was then performed at 42 °C for 1 h. The cDNA samples were kept at −20 °C until analysis by quantitative real-time PCR.

#### 2.8.3. Quantitative Real-Time PCR

The studied primers were designed using Primer3plus soft-ware (Table 1). Each primer-pair was tested and showed an efficiency greater than 95%. Real-time qPCR was performed using GoTaq^®^ qPCR Master Mix kit (Promega). Each reaction mixture was made up of 1 µL of cDNA sample, 2 µL of specific primer pair mix (200 µM each) and 17 µL of a mix consisted of Nuclease-Free Water and GoTaq^®^ qPCR Master containing SyberGreen fluorescent dye. Real-time PCR reactions were carried out in a Mx3000P^®^ qPCR system (Stratagene), and the amplification program was one cycle at 95 °C for 10 min, then 45 amplification cycles at 95 °C for 30 s, 60 °C for 30 s and 72 °C for 30 s. The specificity of the amplifications was checked using the dissociation curve of the PCR products. The dissociation curve was acquired by following the SYBR Green fluorescence level during a gradual heating of the PCR products from 60 to 95 °C.

For each gene, the cycle thresholds (Ct) were obtained from the software MxPro™ qPCR. Two reference genes (*rpl7* and *ef1α*) were used, and the level of gene transcription was normalized with the mean of Ct value of reference genes according to the method of 2^∆∆Ct^ [41]. Induction (>2) or repression (<0.5) factors were determined by the ratio of transcription levels of each condition and the control. The list of studied genes and primer pairs are presented in Table 1.

### 2.9. Statistics

Each condition was carried out in 3 independent replicates. Results are presented as mean ± SD (standard deviation). Normality of data distribution was verified by the Shapiro-Wilk test (*p* < 0.01), and the homogeneity of variances by the Levene test (*p* < 0.05). When data followed a normal distribution, a one-way ANOVA analysis was used (*p* < 0.05) followed by the Tukey post-hoc test. If normality was not met, the non-parametric test of Kruskal-Wallis (*p* < 0.05) was used. All statistical analysis was performed using R software.

## 3. Results

### 3.1. Condition of Exposure

Concentrations of CPF (chlorpyrifos), GLY (glyphosate) and Cu were analyzed at each water change at T_0_, T_24_, and T_48_ in the different treatments to estimate the losses of the compound (Table 2). To analyze the complexes between Cu-GLY in solution, GLY was treated with a metal complex EDTA (ethylene diamine tetra-acetic acid), and Cu was acidified with HNO_3_. In all conditions, the measured concentrations of CPF were inferior to the nominal concentrations at T_0_. For the condition B, all CPF concentrations were below the detection limit. For the two other conditions C and D, CPF concentration strongly declined after a few hours due likely to compound sorption to exposure unit. In the case of GLY, measure concentration was superior to the nominal concentrations at T_0_, and slight decreases were observed at T_24_ and T_48_, but always higher than the nominal concentration. For Cu, the measured concentration was below the quantification limit for the B condition but was very close to the targeted concentrations for conditions C and D at T_24_. In contrast to the two other compounds, Cu concentration increased over time.

### 3.2. Embryonic and Larval Survival

Dissolved oxygen varied from 84% to 94.6% throughout the embryonic and larval exposure. After 3 weeks exposure to the mixture of pesticides, no significant mortality was observed in embryos and larvae in any of the conditions studied (Table 3). Embryonic and larval survival reached more than 95% in all conditions. Hatchability was high, and more than 90% of embryos succeeded in hatching. No significant differences between conditions were observed for the duration of embryonic development.

### 3.3. Phenotypic Effects

Biometric measurements included total size, head size and yolk sac area. No significant alterations were observed in biometrics for larvae exposed to mixtures of CPF, Cu and GLY compared to non-exposed larvae (Table 3). Similarly, no significant increases of developmental anomalies were noted on larvae at the end of the three weeks exposure. Percentages of abnormal larvae were 15.0 ± 2.8, 23.7 ± 3.6, 15.0 ± 7.8 and 22.8 ± 11.3% for control and conditions B, C and D, respectively (Table 3).

### 3.4. Swimming Behavior

**Figure 1:**
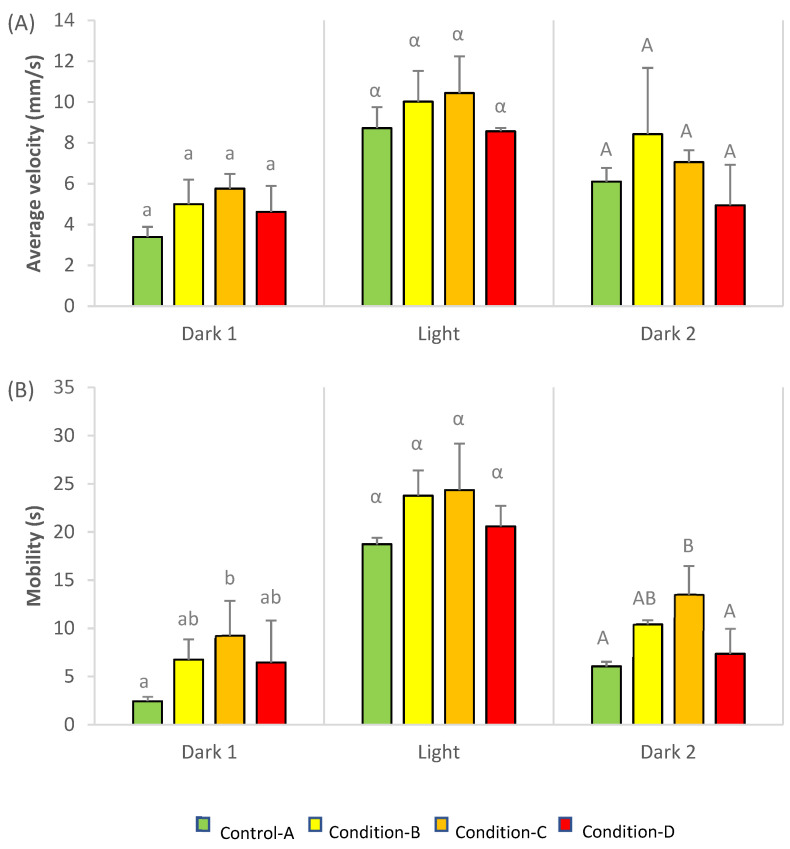
Velocity average (mm/s) (**A**) and cumulative time of mobility (s) (**B**) of rainbow trout larvae following exposure to a mixture of three pesticides, copper, glyphosate and chlorpyrifos. Different letters at the top of the bars indicate significant differences between conditions. Values represent Mean ± SD (*n* = 3, ANOVA, *p* < 0.05).

Average velocity (mm/s) and mobility (s) of rainbow trout larvae subjected to light stress are presented in Figure 1. Average velocity did not show any significant difference between conditions, and only a slight increase between conditions A and C (*p*-value = 0.052) was observed at the basal period (dark 1, Figure 1A). On the other hand, larvae exposed to condition C displayed a significant increase in their mobility compared to control larvae (*p* < 0.05) in both dark periods (dark 1 and 2, Figure 1B). In contrast, no significant differences were observed when larvae were subjected to light stress, due to their high variability.

### 3.5. DNA Damage

**Figure 2:**
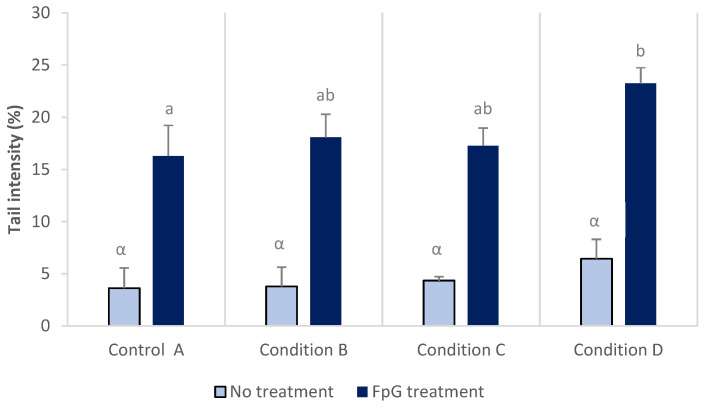
DNA damage in blood cells from rainbow trout larvae after exposure to a mixture of three pesticides (copper, glyphosate and chlorpyrifos), with- and without treatment with the Fpg enzyme. Different letters at the top of the bars indicate significant differences between conditions. Absence of letters indicates no significant differences (Mean ± SD, *n* = 3, ANOVA, *p* < 0.05).

Results of comet assay from blood cells are presented in Figure 2 with and without treatment by Fpg enzyme prior the alkaline unwinding of DNA. Fpg treatment was used to increase the sensitivity of the test. No significant differences in DNA damage were detected between conditions with the classical comet assay and tail DNA intensity ranging from 3.6 to 6.4%. When nuclei were treated with Fpg enzyme, a three to four times increase of DNA damage was observed in comparison to results without Fpg treatment for the same exposure condition. In addition, DNA damage was significantly increased in larvae from control (16.2 ± 3.0%) to condition D (23.3 ± 1.5%).

### 3.6. Lipid Peroxidation and Protein Carbonyls

A significant reduction in TBARS levels was observed on larvae from condition D when compared to control (Figure S1A). However, no significant alterations in protein carbonyl contents were observed in larvae from all conditions when compared to non-exposed larvae (Figure S1B).

### 3.7. Gene Expression

Gene expression levels from whole larvae of rainbow trout revealed several up-regulated genes following a three weeks exposure to pesticide mixture (Table 4). Mixtures of GLY, Cu and CPF resulted in a significant induction of genes involved in detoxification (*gst* and *mt1*), DNA repair (*ogg1*), mitochondrial metabolism (*cox1* and 12S) and cholinergic system (*ache*). High variability in gene transcription was observed for genes involved in oxidative stress (*cat*, *sod*) and no significant differences were observed, nor was the gene involved in apoptosis (*bax*) regulated. Strong genetic induction for 5 out of 10 genes was observed in larvae from condition D, indicating the likelihood that a higher concentration in the mixtures can lead to more severe toxic effects.

## 4. Discussion

Numerous reports have shown that most aquatic ecosystems located close to farmland are contaminated with diverse kinds of pesticide, at different concentrations, which can have an impact on aquatic biota [6,9,42]. Commercial pesticide formulations including copper (Cu), chlorpyrifos (CPF) and glyphosate (GLY) are widely used for crop treatments, and are frequently detected in wetlands and streams, usually transported by runoff [11,17,19,43,44]. The interaction between these molecules can lead to changes in their overall toxicity as a function of the synergistic and/or antagonistic effects [5]. Each studied compound has a different mode of action, and its interaction may imply an induction or an inhibition of a specific metabolic pathway [5].

Our results show no significant mortality of embryos and larvae of rainbow trout exposed to low, moderate and highly concentrated mixtures of Cu, CPF and GLY. In previous studies performed with individual pesticides, no mortality was observed in early life stages of rainbow trout exposed to GLY [28] and CPF [34]. However, significant embryonic mortality (about 10%) and a high frequency of half-hatched embryos (25%) have previously been observed for embryos exposed to 20 µg/L of Cu [15]. In this study, the addition of CPF and GLY seems to inhibit the toxicity of Cu for embryos and larvae of rainbow trout. This result could be explained by possible interactions between compounds. Indeed, GLY is known to be a strong chelator of heavy metals such as Cu [45,46]. The functional groups of glyphosate (amine, carboxylate and phosphate) can react with metal ions to form complexes, resulting in a decreased bio-availability of Cu [47,48]. Our findings are consistent with similar studies with freshwater cladocera (*Ceriodaphnia dubia*) and earthworms (*Eisenia fetida*). Indeed, neonates of *C. dubia* were exposed to seven heavy metals, both alone and in binary mixtures with Roundup^®^, and most of the metals displayed less than additive toxicity [48]. In the same study, LC50-48h for *C. dubia* was estimated at 10 µg/L of Cu^2+^, but in the presence of GLY mortality was reduced to 95%. GLY could also affect the bioavailability of metals: for instance, it favors uptake of Hg and decreases uptake of Ag [48]. Zhou [47] tested the interactions between Cu and GLY on the toxicity of earthworm (*Eisenia fetida*). Acute toxicity of Cu for *E. fetida* was calculated at 0.11 mg/L (LC50-48h), but when it was combined with GLY at concentrations of 0, 2 and 10 mg/L, worm mortalities declined from 57%, to 3% and 0%, respectively. The authors observed that the free Cu^2+^ was reduced with the presence of GLY, preventing the accumulation of Cu in worms.

For the swimming behavior of rainbow trout larvae exposed to single pesticides, we observed that GLY (100 µg/L) induced hyperactivity of larvae, increasing their velocity and mobility under light stimulation [28]. Similar observations were obtained by [24,49,50] in different fish species. On the other hand, larvae exposed to CPF (3 µg/L) were significantly less mobile than non-exposed larvae [34]. Comparable results were observed by [51] and [52], usually related to a decreased of acetylcholinesterase (AChE) activity [53,54,55]. In this study, rainbow trout larvae exposed to the mixture of pesticides at low and medium concentrations were significantly more mobile than control larvae. Mobility of larvae exposed to high concentrations of pesticide did not significantly differ from controls, and no differences were observed after light stimulation. Bonifacio [56] exposed adult females of ten spotted livebearer fish (*Cnesterodon decemmaculatus*) for 6 weeks at concentrations of CPF (0.1 to 1 µg/L) and GLY (0.2 to 2 mg/L). They observed that fish exposed only to CPF had reduced swimming activity, but no differences were observed for fish exposed to GLY and binary mixtures. They hypothesized that GLY could decrease the effect of CPF on swimming activity. In another study, the addition of Cu also diminished the impact of CPF on swimming behavior of adult zebrafish (*Danio rerio*) exposed to a mixture of Cu (6.3 to 40 µg/L) and CPF (35 to 220 µg/L) for 24 h [57]. Indeed, decreased swimming activity was observed in zebrafish exposed to the highest concentration of CPF, suggesting that the addition of Cu may block the biochemical and neurological impacts of CPF or modify its uptake by the neuron. Responses in terms of both hyperactivity and hypoactivity were observed on females of *Jenynsia multidentata* exposed for 24 and 96 h to a binary mixture of CPF (0.4 and 4 µg/L) and cypermethrin (0.04 and 0.4 µg/L) [53]. Low mixture concentrations significantly increased their swimming activity in the upper area of the aquaria, and higher mixture concentrations reduced their swimming activity and caused them to favor the bottom of the tank [53]. Alterations of normal behavior may also depend on the ratio and concentrations of pollutants that are present in a mixture. For example, Kienle [51] exposed zebrafish embryos for 11 days to mixtures of CPF, at 0.25 and 1 mg/L, and nickel chloride (NiCl_2_), at 7.5 and 15 mg/L. When fish were exposed to individual compounds, opposing behaviors were observed, i.e., CPF increased their swimming activity while nickel decreased it. Interestingly, when nickel was combined with low concentrations of CPF, the swimming activity of larvae also decreased; but, combined with higher concentrations of CPF, larvae increased their activity. Kienle [51] argued that both compounds have different modes of action, and in a mixture they may act independently of each other. We can therefore suppose that the hyperactivity observed in our larvae may be an effect caused mostly by the dominance of GLY, in accordance with our previous results [28]. However, since there was an absence of significant response to light change for larvae exposed to mixtures of pesticides, we can assume that the presence of Cu and CPF antagonized or reduced the effects of GLY on larvae. Furthermore, numerous studies have documented the relationship between AChE activity and behavioral changes in fish [54,58,59]. In our work, we observed that ache gene expression was up-regulated in all conditions compared to control, suggesting that alterations observed on behavior of larvae could be related to this induction. Indeed, several pesticides, such as chlorpyrifos and glyphosate, have the capacity to inhibit the AChE activity [23,60]. This inhibition may cause acetylcholine accumulation in synapses of cholinergic neurons, leading to deficiency of important functions such as swimming and feeding [61,62].

When embryos of rainbow trout were exposed separately to Cu, GLY and CPF, no significant increase in DNA damage on blood cells was observed after 3-week exposure compared to non-exposed larvae [15,28,34]. Blood cells from larvae exposed individually to GLY and CPF were also treated with the Fpg enzyme (forma-mido-pyrimidine DNA glycosylase), but no significant changes were observed. Fpg is a DNA-based excision repair enzyme that makes it possible to detect and remove lesions related to basic sites (apuric or apyrimidic), alkylation and oxidative damage (8-oxoguanine) induced by Reactive Oxygen Species (ROS) [37]. In this study, the addition of Fpg enzyme reveals additional DNA damage, in particular for larvae exposed to the highest mixture concentration (condition D). DNA damage has previously been detected by the comet assay in various aquatic organisms exposed individually to Cu, GLY and CPF using environmental concentrations [20,21,63,64]. Few studies have examined the genotoxicity effects of mixtures of pesticides on fish. DNA damage was only observed in hemocytes of the freshwater clam *Corbicula fluminea* exposed to a herbicide mixture of Roundup^®^ (2 and 10 mg/L) and atrazine (2 and 10 µg/L) for 96 h, but no genotoxic effects were observed when clams were exposed to the herbicides separately using the same concentrations [65]. In another study, a mixture of endo-sulfan and CPF (0.94 to 1.88 µg/L) also caused significant DNA damage to erythrocytes of fingerlings of Nile tilapia *Oreochromis niloticus*, after a 70-days exposure [66]. The genotoxicity of mixtures of metals has also been studied using the micronucleus assay on erythrocytes of fish, *Synodontis clarias* and *Tilapia nitolica*, exposed to Cu and zinc (Zn) [67]. In this case, micronuclei frequency was significantly increased when fish were exposed to binary mixtures, compared with exposure to individual metals. No significant protein carbonyl content was observed in exposed larvae when compared to control group. However, TBARS levels from larvae exposed to condition D were significantly lower than control ones. Exposure to individual compounds showed a significant reduction in TBARS levels in larvae exposed to 0.1 mg/L of GLY [28], while Cu and CPF alone did not modify TBARS [15,34]. As we hypothesized previously, the absence of increased TBARS levels and protein carbonyls may be the result of an efficient anti-oxidant system, serving to defend against oxidative stress as observed by [27,68,69] in piava (*Leporinus obtusidens*), freshwater catfish (*Channa punctatus*) and *Anguilla anguilla*, respectively.

With regard to gene expression levels, 3-week exposure to all conditions was mainly associated with the up-regulation in the genes investigated, with similar patterns observed in each of the studied conditions. The exposure to the mixture of the three compounds led to the up-regulation of genes involved notably in detoxification, mitochondrial metabolism and DNA repair. Cu, GLY and CPF are well known to produce individually reactive oxygen species (ROS), and the enzymes catalase (CAT) and superoxide dismutases (SOD) protect the cells from oxidative damage caused by ROS. In our study, no significant regulation of cat and cytoplasmic *sodCu/Zn* genes were observed on exposed larvae because of their elevated variability. However, many studies have shown evidence of the capability of these compounds to induce antioxidant gene expression [62,70,71,72]. ROS neutralization could also be completed by mitochondrial *sodMn* or *gpx* (Glutathione peroxidase) via glutathione oxidation, but these two genes were not investigated in our study. Genes involved in detoxification, *gst* and *mt1*, were induced, especially in larvae exposed to the lowest and the highest mixture concentrations. Expression of *gst* (glutathione s-transferase) is usually up-regulated when fish are exposed to xenobiotics. In our study, *gst* expression was significantly increased in larvae exposed to the lowest mixture concentration (condition B), and this could indicate that larvae were able to implement defense mechanisms against low concentrations of pesticides. GST proteins play an important role in detoxifying xenobiotic compounds by catalyzing the conjugation of reduced glutathione (GSH) on primary metabolites from phase I metabolism [73]. *Mt1* gene encodes the metallothionein proteins, usually induced by metal exposure, such as to copper, but it has also an antioxidant role [74]. Metallothionein proteins have the capacity to bind xenobiotic heavy metals through their thiol groups, providing protection against metal toxicity and oxidative stress [74]. In a previous study using Cu [15], rainbow trout larvae showed a down-regulation of *mt1*, *mt2* and *gst* genes after 3-weeks exposure. However, no significant changes of *gst* gene expression were observed on larvae exposed individually to GLY and CPF [28,34]. Therefore, we can presume that the induction of these genes is the consequence of an additive effect over these functions on larvae exposed to the mixture of pesticides. In our study, a significant induction of *cox1* (cytochrome c-oxidase subunit 1) and 12S gene expression was observed in all conditions compared to control larvae. These up-regulations were even stronger in larvae exposed to the highest mixture concentrations (condition D). An induction of these genes could reveal an increased mitochondrial number per cell and a possible mitochondrial electron transport chain disruption from the lowest mixture concentration tested. The mitochondria is involved in the production of energy for cellular metabolism by synthesizing ATP (adenosine triphosphate). Since the mitochondria is a major source of ROS (superoxide and hydrogen peroxide) through the disruption of the electron-transport chain [75], the up-regulation of *cox1* could denote an increased demand for energy to fight the effects of toxicants. Furthermore, the putative increase of mitochondria could also stimulate ROS production [75,76]. The observed induction of *cox1* and *12S* genes could mean a high-energy demand, in the form of ATP, to defend cells against ROS production or to repair cellular damage by pesticides. This demand of energy may even be required for functions that we have not considered in this study. We also observed, in all conditions, an induction of DNA repair gene expression, ogg1, which is responsible for the removal of 8-oxoguanine as a result of ROS production. The over-expression of *ogg1* (X34.8) on larvae exposed to the highest mixture concentration is probably related to the significant induction of DNA damage that we observed on blood cells after treatment with Fpg enzyme. Consequently, DNA lesions observed in blood cells were likely to be related to oxidative damage. The up-regulation of DNA repair (*ogg1*) and detoxification (*gst* and *mt1*) gene expression may indicate that defense mechanisms were effectively implemented to avoid the effects of ROS production in cells and DNA. This could relate to high demand for energy, with up-regulation observed for 12S and *cox1* gene. However, to validate this relationship, future research could focus on the expression of the mitochondrial superoxide dismutase gene (*sodMn*), which transforms the superoxide anion into hydrogen peroxide, and the expression of glutathione peroxidase (*gpx*), which detoxifies the hydrogen peroxide into water.

It was not clear whether the observed gene regulation in larvae was a consequence of a toxic effect or an adaptation effect. However, since no significant toxic effects were observed on exposed larvae (e.g., mortality, lipid/protein oxidation), we considered that the significant gene up-regulation was mostly an adaptive effect triggered to deal with toxic exposure. At higher mixture concentrations, energy demand increased and the DNA repair, mt1 and ache gene expression greatly increase. This indicates a defense mechanisms induction to prevent toxic effects resulting from increased exposure to chemicals. When comparing the pattern of gene expression from the present work with previous work done with individual exposure to Cu, GLY and CPF [15,28,34], we could consider that the joint action of the three pollutants had an additive or a synergistic effects since the observed effects of the mixture were stronger than the isolated substances.

## 5. Conclusions

Sub-chronic exposure to a mixture of copper, glyphosate and chlorpyrifos on rainbow trout embryos can induce hyperactivity and DNA damage at the higher tested concentrations. In addition, several genes were up-regulated, especially those involved in detoxification, mitochondrial metabolism, cholinergic system and DNA repair. The up-regulation of *gst*, *ogg1*, *mt1*, *cox1* and *12S* gene expression suggests that exposure to the mixture of the three pesticides at realistic environmental concentrations promotes cellular defense mechanisms, indicating the induction of adaptive responses which could limit the occurrence of more severe effects. Our results provide new information about the spectrum of effects and the mechanisms involved in cellular response of rainbow trout embryos exposed to an environmentally relevant mixture of pesticides.

## Figures and Tables

**Table 1:** Accession number and specific primer pairs for the *Oncorhynchus mykiss* used in our study.

Gene	Accession Number	Primer (5′–3′)
*rpl7*	NM_001160672.2	GGTCGCTCTCACAGACAACA ^a^ TTATGTCCGTCCCTCTGGGT ^b^
*cat*	FJ226382.1	CAGGTGTCTTTCTTGTTCAG ^a^ GTCCAGGATGGGAAGTTGC ^b^
*sod Cu/Zn*	NM_001124329.1	TGATTGGGGAGATCTCGGGT ^a^ CGGGTCCAGTGAGAGTCAAC ^b^
*gst*	BT073173.1	ATTTTGGGACGGGCTGACA ^a^ CCTGGTGCTCTGCTCCAGT ^b^
*cox1*	KP013084.1	TCGTTTGAGCCGTGCTAGTT ^a^ CTTCTGGGTGGCCGAAGAAT ^b^
*12S*	KY798500.1	GCGCCAGCTTAAAACCCAAA ^a^ GCCCATTTCTTCCCACCTCA ^b^
*ogg1*	XR_002474791.1	CTGATGGACAAGGCCAGTGT ^a^ GTAAGGACCCCATGGCTGTC ^b^
*rad51*	XM_021612309.1	AGGCTGGAGGAGGACATCAT ^a^ GTATTTGAGGGTGGCAGCCT ^b^
*bax*	BT074328.1	CAGAAAACCCAGGGAGGCAT ^a^ AGAACACATCCTGGGCACAG ^b^
*mt1*	M18104.1	GTGGATCCTGCAAGTGCTCA ^a^ GTAATGCACCAGGCCTCACT ^b^
*ache*	XM_021577686	AGGAGGGTTCTACAGCGGAT ^a^ TATCCTGGACCCACTGGAGG ^b^

^a^ Forward primer ^b^ Reverse primer.

**Table 2:** Nominal and measured concentrations of chlorpyrifos (CPF), copper (Cu) and glyphosate (GLY) for each condition. Concentrations of samples were analyzed at T_0_, T_24_ and T_48_.

	CPF (µg/L) ^1^		Cu^2+^ (µg/L) ^2^		GLY (µg/L) ^3^		
	Nominal Concentration	Measured Concentration	Nominal Concentration	Measured Concentration	Nominal Concentration	Measured Concentration	
				Acidified (HNO_3_)		No EDTA	EDTA
Condition B	0.1	T_0_ < 0.04	0.67	T_0_ < 1.1	33.3	T_0_ 63.3	64.7
		T_24_ < 0.04		T_24_ < 1.1		T_24_ 67.5	49.2
		T_48_ < 0.04		T_48_ < 1.1		T_48_ 55.4	42.2
Condition C	0.3	T_0_ 0.18	2.0	T_0_ 2.03	100	T_0_ 160.0	175.8
		T_24_ < 0.04		T_24_ 4.16		T_24_ 140.0	168.0
		T_48_ < 0.04		T_48_ 4.81		T_48_ 154.0	137.25
Condition D	3.0	T_0_ 1.63	20.0	T_0_ 19.63	1000	T_0_ 1345.0	1785.0
		T_24_ 0.1		T_24_ 26.32		T_24_ 1645.0	2022.5
		T_48_ 0.05		T_48_ 26.65		T_48_ 1775.0	1790.0

^1^ Limit of quantification: 0.13 µg/L, limit of detection: 0.04 µg/L. ^2^ Limit of quantification: 1.1 µg/L, limit of detection: 0.1 µg/L. ^3^ Limit of quantification: 30, 600 and 6000 ng/L.

**Table 3:** Viability and developmental anomalies of early life stages of rainbow trout exposed to a mixture of three pesticides, copper, glyphosate and chlorpyrifos. Values represent Mean ± SD. No significant differences were observed, *n* = 3, ANOVA.

	Control A	Condition B	Condition C	Condition D
Acute toxicity				
Embryo viability (%)	98.7 ± 2.3	96.8 ± 3.4	96.4 ± 1.5	94.2 ± 4.1
Larval viability (%)	97.7 ± 2.1	94.5 ± 4.8	96.0 ± 3.2	95.2 ± 4.9
Cumulative viability (%)	96.4 ± 0.8	91.7 ± 7.3	92.6 ± 3.1	89.5 ± 3.5
Hatching rate (%)	95.6 ± 2.0	92.0 ± 4.6	92.0 ± 4.8	90.2 ± 4.1
Sub-lethal toxicity				
Duration of development (DD)	301.1 ± 2.2	294.9 ± 12.2	305.7 ± 1.1	309.9 ± 8.3
Total length (mm)	19.5 ± 0.3	19.6 ± 0.2	19.8 ± 0.2	19.1 ± 0.2
Head length (mm)	4.5 ± 0.0	4.5 ± 0.1	4.6 ± 0.0	4.3 ± 0.2
Ratio of head/body length (%)	23.2 ± 0.1	23.1 ± 0.2	23.1 ± 0.1	22.4 ± 0.7
Area of yolk sac (mm^2^)	10.8 ± 0.2	10.1 ± 0.4	10.4 ± 0.5	10.5 ± 0.1
Abnormalities (%)				
Total	15.0 ± 2.8	23.7 ± 3.6	15.0 ± 7.8	22.8 ± 11.3
Oedemas	7.5 ± 8.4	12.7 ± 6.2	9.3 ± 5.6	11.9 ± 6.5
Spinal	7.5 ± 8.4	16.6 ± 11.2	12.9 ± 11.3	16.5 ± 14.8
Craniofacial	1.8 ± 3.2	5.4 ± 5.3	11.3 ± 4.5	14.0 ± 4.5
Haemorrhages	7.6 ± 6.6	0.0 ± 0.0	9.3 ± 5.6	11.9 ± 6.5

**Table 4:** Transcription levels of whole larvae of rainbow trout after 3 weeks of exposure to a mixture of copper, glyphosate and chlorpyrifos. Data was expressed as induction (above 2) or repression (below 0.5) factors compared to the control condition. Asterisks (*) refer to significant differences compared to control condition (*n* = 3, ANOVA, *p* < 0.05). /: identical to control.

Gene	Condition B	Condition C	Condition D
*cat*	/	/	/
*sod*	/	/	/
*gst*	2.5 *	/	/
*cox1*	5.3 *	3.8 *	23.8 *
*12s*	4.9 *	5.2 *	32.4 *
*ogg1*	4.4 *	6.3 *	34.8 *
*rad51*	/	/	/
*bax*	/	/	/
*mt1*	9.4 *	/	12.7 *
*ache*	4.5 *	7.0 *	36.7 *

## Data Availability

Data can be requested from the corresponding author.

## Supplementary Materials

The following are available online at https://www.mdpi.com/article/10.3390/toxics9080174/s1, Figure S1: Content of lipid peroxidation expressed as nanomoles of TBARS/mg of protein (A), and content of protein carbonyl expressed as nanomoles of carbonyl/mg of protein (B) in whole body of rainbow trout larvae exposed to a mixture of thee pesticides, copper, glyphosate and chlorpyrifos.
