# Supplementary material for: Environmentally Relevant Mixture of Pesticides Affect Mobility and DNA Integrity of Early Life Stages of Rainbow Trout (Oncorhynchus mykiss)
Source: Toxics. 2021 Jul 22;9(8):174. doi: 10.3390/toxics9080174 (PMC8402510; doi:10.3390/toxics9080174)
Supplement: Supplementary file 1 [file toxics-09-00174-s001.zip › toxics-1292570-supplementary.pdf]

toxics

MDPI

Article

Supplementary material: Environmentally Relevant Mixture of Pesticides Affect Mobility and DNA Integrity of Early Life Stages of Rainbow Trout (Oncorhynchus Mykiss)

Shannon Weeks Santos, Jérôme Cachot, Bettie Cormier, Nicolas Mazzella, Pierre-Yves Gourves, Christelle Clérandeau, Bénédicte Morin and Patrice Gonzalez

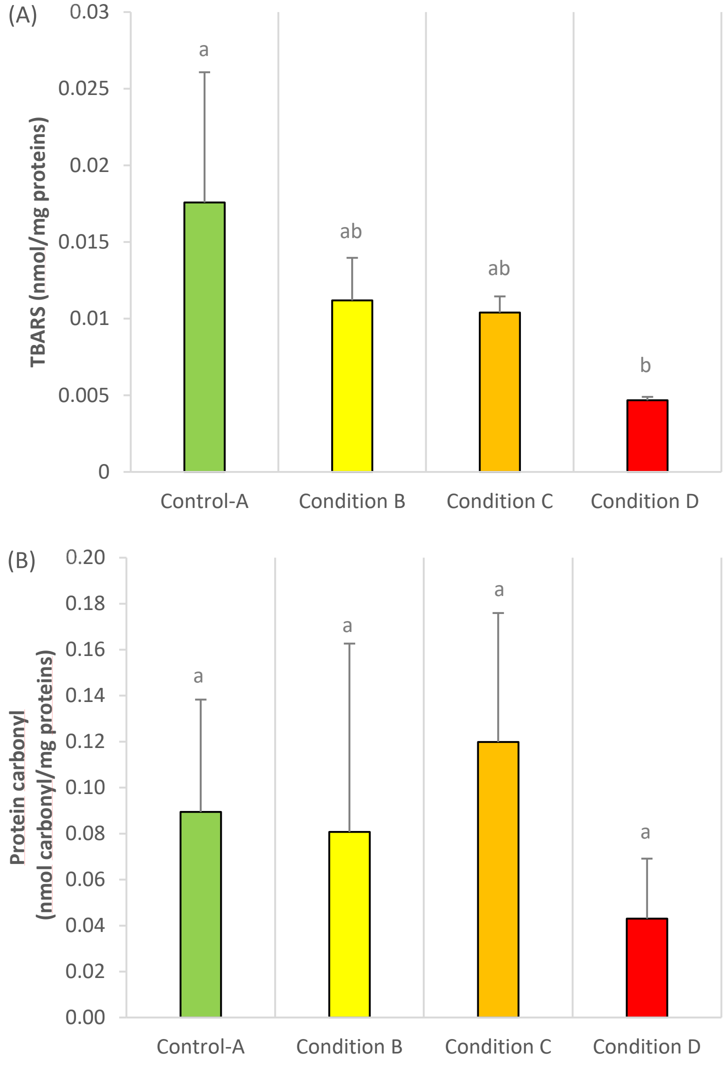

Figure S1. Content of lipid peroxidation expressed as nanomoles of TBARS/mg of protein (A), and content of protein carbonyl expressed as nanomoles of carbonyl/mg of protein (B) in whole body of rainbow trout larvae exposed to a mixture of thee pesticides, copper, glyphosate and chlorpyrifos. Different letters at the top of the bars indicate significant differences between conditions. Absence of letters indicate no significant differences. Values represent Mean ± SD (N = 3, ANOVA, $p < 0.05$).
